# *Eryngium foetidum* L. (Apiaceae): A Literature Review of Traditional Uses, Chemical Composition, and Pharmacological Activities

**DOI:** 10.1155/2022/2896895

**Published:** 2022-03-14

**Authors:** Thiara L. M. Rodrigues, Maria E. P. Silva, Ely S. C. Gurgel, Mozaniel S. Oliveira, Flavia C. A. Lucas

**Affiliations:** ^1^Programa de Pós-Graduação Biodiversidade e Biotecnologia, Universidade Federal do Pará, Belém, Pará, Brazil; ^2^Universidade Federal Rural da Amazônia, Belém, Pará, Brazil; ^3^Coordenação de Botânica, Museu Paraense Emílio Goeldi, Belém, Pará, Brazil; ^4^Universidade do Estado do Pará, Belém, Pará, Brazil

## Abstract

*Eryngiumfoetidum* L. is popularly known as *chicória-do-Pará*, is native to the Amazon region, and is widely distributed in Northern Brazil. It is considered a versatile species due to its diversified uses in ethnomedicine, gastronomy, and pharmaceutical industry. The objective of this study was to review the literature on the traditional uses, chemical composition, and pharmacological activities of *E*. *foetidum* based on information published in national and international scientific articles between the years 2011 and 2021. Literature searches were performed with the combination of the expressions “*Eryngium foetidum* L.,” “chicória-do-Pará,” “traditional uses,” “ethnobotany,” “volatile compounds,” and “essential oil.” The species is widely used as a flavoring condiment in beans, meat, duck, and fish, and in the preparation of tucupi (cassava sap), showing to have great importance for the Amazonian food culture. In traditional medicine, it has analgesic, antibacterial, antiflu, and antipyretic applications. The chemical profile is characterized by the presence of aromatic and aliphatic aldehydes, mainly (2E)-2-dodecenal in leaves and 2,3,4-trimethylbenzaldehyde in roots, in addition to fixed compounds such as carotenoids, flavonoids, and phenols. These compounds have shown diverse biological activities and potential antibacterial, anthelmintic, and antioxidant applications, confirming their potential for use in folk medicine. Therefore, it is inferred that this aromatic plant has vast potential uses and is an important alternative as a natural resource for the food and pharmaceutical industries in view of its antioxidant capacity and bioactive compounds.

## 1. Introduction

*Eryngium foetidum* L. (Apiaceae), popularly known in Brazil as *chicória-do-Pará*, *coentrão*, *chicória-de-caboclo*, *chicória-da-Amazônia*, and *coentro,* is an unconventional seasoning vegetable and has attracted the interest of researchers due to its wide versatility and growing popularity [1, 2]. One of the important characteristics of the species is the presence of essential oils in its secretory ducts, which are specialized cells present throughout the plant's body rich in bioactive compounds, which add economic value to this plant in international trade and in the perfumery and pharmaceutical industries [3–5].

The peculiar flavor and aroma of *E*. *foetidum* are due to a chemical constituent present mainly in the essential oil of the leaves, mostly found as an aliphatic aldehyde called eryngial [5, 6]. The leaves are widely appreciated as a condiment and used to season everyday foods such as beans, salads, meat, and fish, as well as in the preparation of typical and traditional recipes such as fish stew, *tacacá*, *pato no tucupi* (duck stew made with tucupi (indigenous origin; yellowish broth extracted from the roots of wild cassava, boiled and seasoned with spices such as garlic, chicory, and pepper [7])), and *arroz paraense* (rice with shrimp and other condiments cooked in tucupi) [8, 9].

This species is also used in ethnomedicine, since it has shown potential application in diseases related to the gastrointestinal tract and acts as an antibacterial, analgesic, anti-inflammatory, anthelmintic, anticonvulsant, and anticancer agent, thus proving to have great ethnopharmacological importance for people [10–12]. In this sense, studies have analyzed the applications of *E*. *foetidum* and have pointed to its pharmacological potential, notably Bhavana et al. [3], Forbes et al. [13], Sumitha et al. [14], and Leitão et al. [15], confirming the bioactive and antioxidant potential of this plant.

For Singh et al. [16], the high medicinal and nutritional value of *E*. *foetidum* is due to the presence of a range of metabolites such as aldehydes, carotenoids, phenolic compounds, and anthraquinones. However, the knowledge on the potential use of this plant for the population is restricted to traditional communities and people [2, 17].

In this context, this study aimed to review the literature on the traditional uses, chemical profile, and pharmacological activities of *chicória-do-Pará* and its role in Amazon's gastronomic cultural heritage based on information collected from scientific articles published from 2011 to 2021 to value the potential uses and analyze future prospects of applications of *E*. *foetidum*.

## 2. Methodology

A bibliographic review of documents published between 2011 and 2021 [18] on the subject was conducted. Literature searches with the following terms in English and Portuguese were carried out: “chicória-do-Pará”; “traditional uses”; “ethnobotany”; “volatile compounds”; and “essential oil” associated with “*Eryngium foetidum* L.” and/or “Amazon.” Publications from the last ten years were prioritized. The data search tools were as follows: Google Scholar (https://scholar.google.com.br), CAPES Portal (https://www.periodicos.capes.gov.br/), Web of Science (https://www.web.of.sciencegov.br/), and PubMed (https://pubmed.ncbi.nlm.nih.gov/). Only full-length scientific articles and national and international books were considered; monographs, dissertations, and such were excluded.

We favored full-length articles on the topics of taxonomic description, geographic distribution, agronomy, and ethnobotany or ethnobiology that included *E*. *foetidum* in the list of uses or described the chemical profile and pharmacological applications of the species. The method of categorization of information was adopted for the analysis of scientific productions, grouping key elements to synthesize information, including the dimensions developed in this manuscript [19].

## 3. General Description

### 3.1. Botanical Aspects, Propagation, and Cultural Treatments

*E*. *foetidum* belongs to Apiaceae Lindley (=Umbelliferae Juss.), a family with approximately 400 genera and 4,000 species, occurring mainly in tropical and subtropical regions, with largest distribution in Neotropical regions [20]. In Brazil, 15 genera and 91 species are recognized [21, 22]. According to Boldrini [20], the genus *Eryngium* L. has 250 species distributed in Eurasia, North Africa, North, Central and South America, and Australia. It is composed of herbaceous plants that preferentially colonize terrestrial, rock, and aquatic substrates [21].

**Figure 1:**
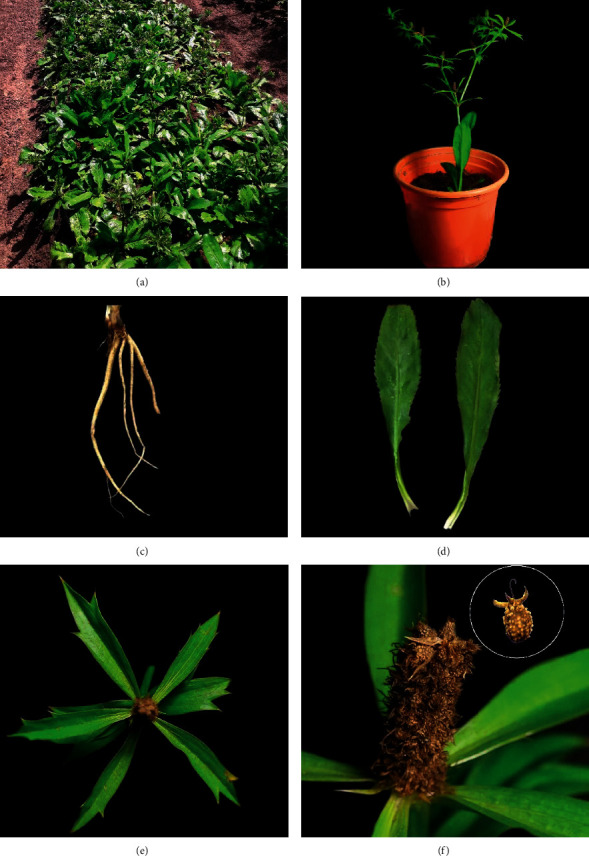
(a) *Eryngium foetidum* L.; (b) in flowerbed; (c) in the vase with leaf and inflorescence; (d) stem and fasciculated root; (e) toothed sheet; (f) bracts, fruit, and seed.

The species is popularly known as *chicória-do-Pará*, *chicória-da-Amazônia*, *coentrão*, and *culantro* [2, 23]. It is widely used as a condiment and in traditional medicine. It is included in the list of species of the Brazilian socio-biodiversity, classified as a rustic tropical vegetable of herbaceous habit, measuring 45 cm in height [22]. The roots are light beige and branched; the stem is straight and cylindrical; and the leaves are basal, spatulate, membranous, glabrous, rosulate, and with a sheath that resembles a channeled petiole. The inflorescences are cymose and can be solitary, paired, formed by a floral axis, with bisexual flowers arranged in small and dense sessile, cylindrical, or ovoid, long-pedunculated capitula. The fruits are globose and scaly [22]. The fruits are indehiscent, densely papillate, white, 1.5–2.5 mm in diameter, and globose to ovoid; mericarps measure ± 1.5–2.5 × 1.5 mm and present tiny, globose, isomorphic papillae on the dorsal face, and cylindrical to oblong ventral face, enlarged at the apex [24].  Figure 1 illustrates the morphological structures of the adult plant.

Propagation of the species occurs through the seeds or reuse of clumps, which can be transplanted to the soil [23]. Seeds are obtained from bolting plants planted in the previous months, which are usually chosen and separated for reproduction. Seedlings and botanical material can be acquired through exchanges between local residents, as this plant is autogamous and produces seeds in abundance, typical of the genus *Eryngium*, thus facilitating the reproduction of the species [24].

According to Gomes et al. [23], the sowing of chicory occurs in nursery/seedbeds with a 15 cm plant-to-plant spacing. Fertilization is basically organic, and using broiler litter, [25] recommend the application of biological charcoal associated with organic fertilizer to obtain greater yield of dry and fresh mass of shoots and roots, providing better development to the plant. In the initial stages of plant growth, it is necessary to keep the soil moist through regular watering; after that, it is necessary to irrigate occasionally [26].

Furthermore, it is important to highlight that the management of the species is reported by 100% of the producers of Cáceres-MT: as the plant develops, early bolting is seen as an obstacle to the productivity of the species because it paralyzes the growth of the plant, mainly reducing the production of leaves. This happens because the plant directs photoassimilates to the development of flowers and seed production. Thus, the pruning of the floral tassel is suggested to redirect nutrients to the production of the leaves to enhance fresh mass yield [23, 24].

### 3.2. Origin, Occurrence, and Distribution

**Figure 2:**
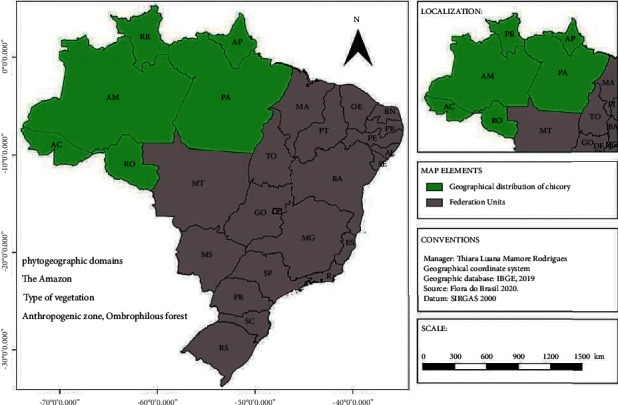
Phytogeographic domains of *E*. *foetidum* L. in Brazil. Source: adapted from Flora do Brasil [22].

*E*. *foetidum* is native to Central and Latin America and occurs in tropical regions, North and Central America, South Asia, the Pacific Islands, Europe, and Southern Africa [4]. It is widely distributed in the Brazilian territory, with occurrences in Acre, Amazonas, Amapá, Pará, Rondônia, and Roraima (Figure 2), with phytogeographic domain in the Amazon [22].

## 4. Traditional Uses

In the last ten years, numerous scientific research articles have cited traditional uses of *E*. *foetidum*, especially in Latin America and Asia. Twenty-one studies mentioned the use of the species in ethnomedicine in the form of tea to treat inflammation, and nine mentioned it with the application in gastronomy to prepare typical foods.

### 4.1. Use in Traditional Medicine

Historical texts written between the 16th and 17th centuries mention *E*. *foetidum* as a plant frequently cultivated in the Brazilian territory, known for its medicinal and culinary uses [2, 27]. Previous studies mention chicory as an herb of diverse uses, becoming a material and immaterial good for families belonging to traditional communities. Acosta [28], Montes-Rojas e Paz-Conchas [29], and Gonçalves e Lucas [8] say that *E*. *foetidum* is a plant used in folk medicine, considering it an excellent way to treat illnesses.

In South American countries such as Peru, Colombia, and Ecuador, the plant is used to treat diseases and ailments related to the digestive and gynecological tract, such as flatulence [2], diarrhea, indigestion, and stomach pain [30, 31]. The tea of the plant is recommended to treat female reproductive problems, promote menstruation, relieve cramps, treat infertility, and facilitate labor, and it is considered to have aphrodisiac action [28, 31, 32]. Scientific productions also highlight the importance of the use of medicinal plants by different peoples and native populations, as they contribute to maintain plant biodiversity, valuing cultural aspects and local identity [2, 33, 34].

For Rosero-Gómez et al. [2], in the San Antonio de Padua community, Ecuador, the forms of traditional uses of the species are passed on from generation to generation, by parents (37%), grandparents (27%), and even spouses (13%) of the residents. They immerse the entire plant (leaves and roots) in hot water to produce tea to treat digestive tract problems such as an imbalance in the intestinal flora. It is also used in the form of plaster to relieve joint pain, especially in the knees [2]. Traditional knowledge of the medicinal use of chicory points to the potential topical use of the species, and Fongod et al. [17] reported the indigenous knowledge about this plant in the South and Southeast region of Cameroon: the leaves are pressed or ground with a little water and used externally to treat abscesses and boils.

Vásquez et al. [35] recorded the use of *E*. *foetidum* in the form of tea and syrup to fight flu in four riverside communities in Amazonas (São Raimundo, Bom Jardim, Nossa Senhora do Livramento, and Rei Davi). The use of the plant to fight flu was also registered in the Quilombola community Taumara-Açu, located in the municipality of Abaetetuba-PA [36]. Fever, cold, sore throat [30, 37], headache [38], and infection [30, 39] are some of the other conditions treated with chicory, mainly in Amazonian communities.

Other important diseases treated with chicory include skin changes, disorders of the nervous and respiratory system [40], hepatitis [41, 42], seizures, malaria, sexual impotence, and kidney problems [31]. It is also reported to be used to alleviate anxiety [37]. In Colombia, the whole plant is used as antiscorbutic, antirheumatic, antiseptic, against vomiting, nausea, headache, and hemorrhage. Another form of use is in the preparation of baths and in the treatment of smallpox and gonorrhea [31]. In addition, it is used as the ethnomedicine by the traditional people of the form of tea for the treatment of stomach pains [34, 43].

The human experience of the use of *E*. *foetidum* in ethnomedicine brings together repertoires of local knowledge for the cure of diseases and ailments, legitimizing the value of traditional knowledge in communities. Dialogues between memory and tradition configure the transmission of information to descendants, and older women are remarkably the holders of knowledge about the use of the plant [8]. Chicory is recognized as a material and immaterial heritage for agricultural families, communities, and traditional peoples, as it is a natural alternative to help maintain human health.

### 4.2. Food Use

When talking about the food use of chicory, the *Ver-o-Peso* market in Pará stands out as a representative of the cultural heritage of the state. It is one of the largest open-air markets in Latin America, with points of sale of delicacies from the territory of Pará that make up one of the largest market places of bio-edible samples in the Amazon [44]. Herbs, spices, and aromas are commercialized, including some regional herbs, symbols of the local cuisine, for example, *jambu* (*Spilanthes oleracea* L.) and *chicória-do-Pará* (*Eryngium foetidum* L.). The latter offers a special flavor and aroma to foods, which comes from its chemical components, especially (2E)-2-dodecenal, known as eryngial [5, 6].

Plants are used as flavoring in various foods, Vilhena et al. [45], Batista and Barbosa [46], and Barros et al. [47] report that *E*. *foetidum* is a food condiment. Gonçalves and Lucas [8] present *E*. *foetidum* as a condiment cultivated in backyards close to the kitchen among the food species used by the traditional peoples of Altamira, Pará, Brazil. It is also considered as a family asset by these people because, in the process of deterritorialization that took place in Belo Monte, river-dwelling families transported plants from their productive areas to new homes, and chicory was carried in their luggage along with other plants and personal objects, [48]. This fact reveals the interaction and cultural and ethnic aspects as well as the traditional culinary value of the species.

Vilhena et al. [45] mention that in the shopping centers of Belém-Pará, typical regional food recipes such as *tacacá* (indigenous origin; prepared with “tucupi” and cassava starch, served with dried shrimps and “jambu” [44]), *vatapá* (typical of the Afro-Brazilian cuisine; it is usually prepared with corn starch, milk, salted shrimp, palm oil, coriander, chicory, onion, garlic, green pepper, and tomato [49]), *caruru* (African origin; widely consumed in the northern region of Brazil; it is a stew made from cassava flour, dried shrimp, palm oil, pepper, chicory, coriander, onion, and tomato [49]), and *maniçoba* (indigenous origin; dish whose main ingredient is “maniva,” i.e., cassava leaves ground and cooked with pork, salted beef, and sausages; it resembles “feijoada,” with cassava leaves in the place of beans [50]) are sold for appreciation of flavors. Due to the influence of the local food culture, *E*. *foetidum* is used in recipes to add flavor and preserve food. Dorneles and Chaves [51] highlight *E*. *foetidum* as an indispensable spice in the preparation of *tacacá*, known as a food of indigenous origin prepared with cassava products such as *tucupi* and starch (derived from a type of indigenous soup called *mani poi*; it is made from the starch that is extracted from the cassava; it is also known as tapioca gum and dry gum [50]), along with salted shrimp and *jambu* leaves (a flowering herb typical of Northern Brazil). It is served hot in a traditional indigenous utensil: the *cuia* (type of gourd), supported by a straw basket. *Pato no tucupi* is mainly consumed in Círio de Nazaré, seasoned with salt, garlic, and black pepper, cooked in *tucupi*, with the addition of *E*. *foetidum*, *jambu*, and basil, and served with white rice, cassava flour, and habanero pepper sauce [52] (typical of the Afro-Brazilian cuisine; it is usually prepared with corn starch, milk, salted shrimp, palm oil, coriander, chicory, onion, garlic, green pepper, and tomato) [49].

Some preparations are mujica [7], *tacacá* [51] *caruru*, and *caldeirada de peixe no tucupi* [9]. Fish dishes and *moqueca* (saltwater fish stew in coconut milk) [53], and *pato no tucupi* [54] include chicory as a condiment. In Asia, chicory is used in soups, noodle dishes, and curry (spice mix) [6]. There are a greater number of studies from Latin American countries that mention the use of chicory as a condiment for food seasoning [7, 27, 46, 54]. The traditional uses of *E*. *foetidum* are summarized in Table 1.

## 5. Chemical Profile

In the literature, 22 articles on this theme were found in the different databases surveyed.

### 5.1. Volatile, Fixed Compounds, and Minerals

Some essential oils are products of secondary metabolism, normally produced by aromatic plants. They are defined as complex mixtures and have high added value due to their applications in the pharmaceutical industry [5, 15].

The chemical profile of the essential oils from *E*. *foetidum* is characterized by aromatic and aliphatic aldehydes. The major constituent, (2E)-2-dodecenal, known as eryngial, was first reported in the literature by Koolhaas in 1932 and is the substance responsible for the peculiar flavor and aroma of the species [57]. Note that the chemical composition of the chicory leaf and root is similar, because Chandrika et al. [58], Thomas et al. [1], and Rodrigues et al. [5] identified (2E)-2-dodecenal in the leaves and roots of *E*. *foetidum*, in addition to 13-tetradecenal, *trans*-2-tetradecenal, 2,3,4-trimethylbenzaldehyde [5], 2,4,5-trimethylbenzaldehyde, dodecanal [1], 1, trimethylbenzaldehyde, *τ*-cadinol, and *α*-cadinol [58].

Chandrika et al. [58], Thomas et al. [1], and Rodrigues et al. [5] analyzed the chemical profile of the vegetative organs of *E*. *foetidum* in India, Nigeria, and Brazil (Pará), respectively, and reported (2E)-2-dodecenal as the major constituent present in the leaves, while the main elements in the roots were 2,3,4-trimethylbenzaldehyde (Brazil) and 2,4,5-trimethylbenzaldehyde (India and Nigeria). Among the vegetative organs, the moisture of the leaves can vary from 10.33% to 87% according to the methodology used [5, 15, 59]. For the identification of the chemical constituents of chicory, gas chromatography-spectrometry analysis is the most used method [1, 6, 58, 60, 61].

The methods used for extraction of the essential oil from chicory include the hydrodistillation and steam distillation method. Hydrodistillation has been the most used, reported in the works by Jaramillo et al. [60], Ngang et al. [61], Sumitha et al. [14], Chandrika et al. [58], Thomas et al. [1], Rodrigues et al. [5], and Castro-Alayo et al. [62], and steam distillation is used for extraction. With these methods, levels from 2.8% [58] to 7.65% [1] of (2E)-2-dodecenal were found in the roots and between 14.3% [58] and 50.62% [61] in the leaves. There was also a qualitative difference between the identified compounds: Castro-Alayo et al. [62] cited (Z)-13-octadecenal, *α*-pinene, *m*-cymene, and *o*-cymene components that are not mentioned in other studies as the major compounds.

Among the constituents identified in the essential oil of *E*. *foetidum* leaves reported in the works, only three compounds do not differ, namely (2E)-2-dodecenal, the most often cited in the literature, found at percentages varying from 50.62% [61] to a minimum of 14.3% [58]; dodecanal, found at maximum values of 14.59% and minimum values of 4.7% [1]; and Ngang et al. [61] with approximate levels of 11 and 10.77%, respectively. Therefore, there are no qualitative differences between the essential oils of leaves and roots, but there are quantitative differences in terms of percentage. The other major compounds listed in Table 2 appear only once in each study.

In India, 93 compounds were detected in leaf and root samples of *E*. *foetidum* from two localities (Port Blair and Nadugani). (2E)-2-dodecenal (2.9%), trimethylbenzaldehyde (16.5%), dodecanal (4.7%), and caryophyllene oxide (2.6%) were found in the leaves of the plants from Port Blair. The oil from leaves of the samples from Nadugani was rich in the constituents trimethylbenzaldehyde (14.3%), dodecanal (3.3%), (2E)-2-dodecenal (14.3%), *τ*-cadinol (5.1%), and *α*-cadinol (6.9%). The main compounds of the oil from the roots of plants from Port Blair were isomeric trimethyl benzaldehyde (57.0%) and dodecanal (2.3%). In turn, the oil from the roots of plants collected in Nadugani had a composition similar to that of the leaves, with the addition of (2E)-2-dodecenal (2.8%), *τ*-cadinol (7.3%), and *α*-cadinol (10.2%) as main compounds [58].

**Figure 3:**
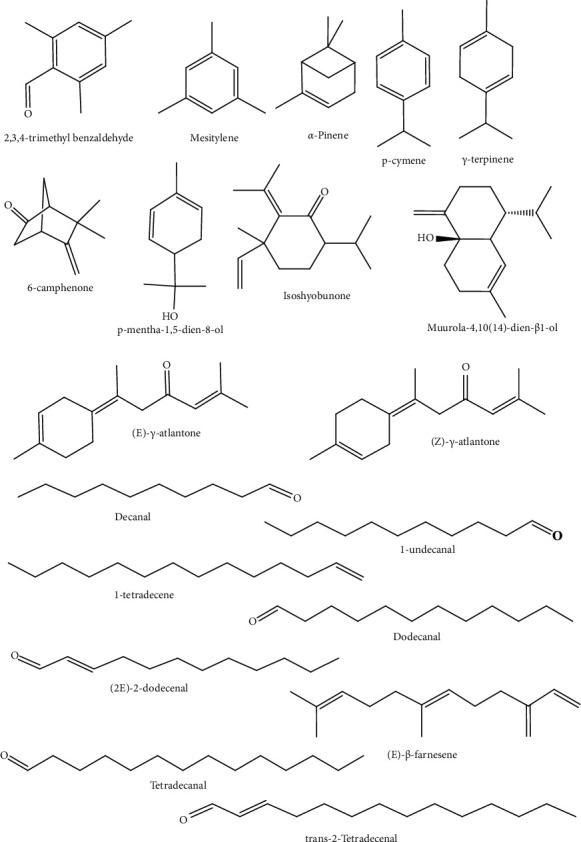
Structures of chemical compounds identified in essential oils of *Eryngium foetidum* L. adapted from Rodrigues et al. [5].

Similar data were found in leaf samples of *E*. *foetidum* in Colombia, which showed a high percentage of aldehydes ((2E)-2-dodecenal, 5-dodecene, tetradecanal, tetradecenal) and aromatics (2,4,6-trimethylbenzaldehide, 3,4,5-trimethylphenol) [60]. The essential oil of leaves of *E*. *foetidum* has a high antioxidant capacity, being therefore an important source of natural biocompounds [16]. It is noted that essential oils from *E*. *foetidum* are marked by aromatic and aliphatic aldehydes: (2E)-2-dodecenal in leaves and 2,3,4-trimethylbenzaldehyde in roots. The chemical compounds present in the essential oil of *E*. *foetidum* are shown in Figure 3.

It can be observed that the chemical composition is variable among species of the genus Eryngium. In *Eryngium bungei* Boiss, the major components found in the hydrodistilled oil were borneol (44.4%), isobornyl formate (14.7%), isoborneol (9.2%), 1,8-cineole (9.1%), and camphor (7.9%) [64]. In *Eryngium caeruleum* Bieb, the oil was mainly composed of limonene (25.42%), cyclobuta[1, 2 : 3, 4]dicyclooctene-hexadecahydro (22.24%), and *δ*-2-carene (16.19%) [65]. It is important to emphasize that none of these compounds are mentioned as the major compound of *E*. *foetidum*.

When analyzing the chemical profile of *Eryngium campestre* L. and *Eryngium amethystinum* L., Cianfaglione et al. [66] detected similar compounds, including sesquiterpene hydrocarbons, with germacrene D, followed by allo-aromadendrene, *β*-elemene, spathulenol, and ledol. These compounds were not found in *E*. *foetidum*. However, the chemical composition of species of the genus Eryngium is not restricted to volatile organic compounds and works such as that found by Paun et al. [67], who highlights that *Eryngium planum* L. contains mainly flavonoids, especially rutin, a compound also present in *E*. *foetidum*, cited by Chandira and Jaykar [68], Singh et al. [16], Lingurajo et al. [12], Campos et al. [69], and Nguyen [70]. However, alkaloids and anthraquinones were also found, and these constituents are rarely found in *E*. *foetidum* [71, 72]. Swargiary et al. found that *E*. *foetidum* contained high content of carbohydrates (174.72 ± 1.72 *μ*g/mg) and proteins (65.58 ± 5.26 *μ*g/mg), in addition to vitamins B12 and C, or ascorbic acid, present at high concentration in the leaves (14.17 ± 1.17 *μ*g AAE/mg). This vitamin is antioxidant and an important free radical catalyst, justifying the nutritional value of the plant [73–75]. The main constituents of the chemical profile of *E*. *foetidum* are summarized in Tables 2 and 3.

In addition to organic compounds, other studies have shown that *E*. *foetidum* has in its composition the presence of fixed minerals, among others. As found in the study by Singh et al. [59], *E*. *foetidum* presented high concentrations of crude fiber (maximum of 6.32% and minimum of 0.51%); the protein content ranged between 5.25% and 0.13%, and crude leaf fat ranged between 1.95% and 0.06%. Other minerals such as potassium (K), phosphorus (P), cobalt (Co), manganese (Mn), copper (Cu), sodium (Na), zinc (Zn), calcium (Ca), iron (Fe), vanadium (V), and magnesium (Mg) were also found.

## 6. Pharmacological Activities

Nineteen studies on the use and pharmacological application of *E*. *foetidum* were found, with greater emphasis on antibacterial, antioxidant, antifungal, and anti-inflammatory activities.

### 6.1. Antibacterial Activity

In this section, six studies published between the years 2011 and 2021 were found in the databases. India stood out with the majority (four) of the studies on the theme.

*Helicobacter pylori* is a bacterium that affects the stomach. About 50% of people test positive for the presence of this pathogen and 20% develop related gastroduodenal diseases. Mabeku et al. [71] conducted *in vitro* and *in vivo* tests in rats to measure the reduction in the bacterial load of six strains using methanol extracts of *E*. *foetidum* (500 mg/kg). The extracts resulted in an abundance of metabolites and antipathogenic properties both *in vitro* and *in vivo*, with efficiency similar to ciprofloxacin. Panda et al. [79] showed the antibacterial effect of this herb through the use of methanolic extracts, acting in the minimum inhibitory concentrations (≥12 mm) against *Bacillus cereus*, *Staphylococcus aureus*, *Escherichia coli*, *Salmonella typhimurium*, *Shigella sonnei*, *Shigella dysenteriae*, *Shigella flexneri*, and *Vibrio cholerae*.

For Begum et al. [80], the nontoxic and economical biosynthesis technique of ZnO nanoparticles (NPs) used to assess the antibacterial potential of herbs is an ecologically clean and environmentally acceptable technique. The extract of *E*. *foetidum* leaves was analyzed through this characterization by these authors, and they found that the biosynthesized ZnO NPs were an excellent agent against *E*. *coli*, *Pseudomonas aeruginosa*, *S*. *aureus subsp. aureus*, and *Streptococcus pneumoniae* [80]. In addition, the combination of chicory with other popular medicinal plants from Assam, India, presented enhanced antibacterial and antifungal effects [72]. There was a synergistic positive effect of *E*. *foetidum* + *Bacopa monnieri* against *S*. *typhimurium*. The medicinal characteristics of these plants may be due to the presence of alkaloids, flavonoids, tannins, steroids, saponins, and phenols [72].

The methods of solvent extraction from leaves have an influence on the quantification of active principles and the assessment of potential activities of plants. For example, Dalukdeniya and Rathnayaka [78] reported that the methanol and chloroform extracts had high antibacterial activity against *S*. *pneumoniae*, *Listeria monocyatogenes,* and *S*. *aureus*, while the water extract showed greater activity against *S*. *typhimurium*.

Linguarajo et al. [12] confirmed the inhibition of the bacteria *E*. *coli* (zone of inhibition 17 mm), *P*. *aeruginosa* (zone of inhibition 28 mm), *Bacillus subtilis* (zone of inhibition 20 mm), *S*. *aureus* (zone of inhibition 25 mm), and fungus *C*. *albicans* (zone of inhibition 18 mm) when using the organic solvent, ethyl acetate, due to its higher concentration of bioactive agents. Therefore, the authors confirmed the microbial activities of *E*. *foetidum* in traditional medicine. The plant is used in Karnataka, India, in the form of tea and plaster for intestinal disorders and wound healing, respectively.

### 6.2. Antioxidant Activity

Four texts about the antioxidant activity of *E*. *foetidum* were registered between 2011 and 2021. It was observed that the locality, phenological phase, the analyzed part of the plant, and the extraction method influence the assessment of the oxidative potential of the species.

Certain bioactive compounds present in the essential oils of plants have the function of protecting cells from the harmful effect of reactive oxygen species and are therefore called *natural antioxidants*. They delay the degeneration processes related to aging and diseases such as cancer, cardiovascular disease, and neurodegenerative disorders [16]. Singh et al. [16] quantified the extraction of chemical compounds from *E*. *foetidum* leaves and found greater concentrations in acetone and methanol solvents, which were rich in polyphenol, tannin, anthocyanin, flavonoids, carotenoids, and ascorbic acid. These findings indicate that this plant has high potential for the pharmaceutical and food industry [16].

Thomas et al. evaluated the antioxidant activity of *E*. *foetidum* leaves, roots, and stems through the free radical scavenging capacity of volatile oils using 2,2-diphenyl-1-picrylhydrazyl (DPPH) and ferric reducing antioxidant power (FRAP) assays. In the DPPH tests, the oils from leaves, stems, and roots presented IC_50_ values of 56 *μ*g/mL, 46 *μ*g/mL, and 54.5 *μ*g/mL, respectively, while in the FRAP test, the leaf oil had the greatest reduction potential among the tested oils. Acyclic aldehydes and aromatic compounds, which are important antioxidant agents, were also detected. Investigations by Nguyen [70], on the total phenolic, flavonoid, and antioxidant capacity of *E*. *foetidum* extract, determined that extraction with 60% ethanol, at 50°C, stirring duration of 30 minutes, particle size of 0.4 cm, and solid-liquid ratio of 1 : 25 g/mL provided the highest total phenolic, flavonoid, and antioxidant capacity. These methods are relevant to obtain the highest concentration of bioactive compounds.

The antioxidant capacity of *E*. *foetidum* is influenced by the growth stage: in the juvenile stage, between 90 and 120 days after germination, there is greater production of bioactive compounds such as polyphenol and flavonoids with antioxidant activity in the leaves [69]. On the other hand, Castro-Alayo et al. [62] observed that in plants from 52 districts of the Amazon region in Peru, there was a variable yield, concentration, and antioxidant activity of the essential oil of chicory. In this region, the most prominent volatile component was *α*-pinene (23.41%) and the content of the essential oil varied among individuals, suggesting a variability associated with geographic location [62].

### 6.3. Antifungal Activity

Lingarajo et al. [12] and Borah et al. [72] evaluated the potential of extracts from *E*. *foetidum* leaves and found antifungal activity for *C*. *albicans*, with high inhibition. This shows that substances produced by the species can be useful to treat diseases caused by this pathogen, such as infections of the gynecological tract. It also ratifies the efficiency of this plant when used for this purpose in traditional communities.

### 6.4. Anti-Inflammatory Activity

Anti-inflammatory action was also reported by Mekhora et al. [81] and Dawilai et al. [76]. Their data suggest a significant role in the suppression of the pro-inflammatory process and a high potential of the plant to be used as a food supplement to reduce the risk of cancer associated with inflammation. Other activities have been reported for the species, such as anticlastogenicity [82], antilarval [14], anticancer [83], antidiabetic [68], and toxicological [84] activities.  Table 3 summarizes the pharmacological activities of *E*. *foetidum* and its bioactive compounds.  Table 4 gathers the information about the pharmacological activities of the extract from *E*. *foetidum* in different microorganisms.

## 7. Conclusion

This study is the result of the collection of 88 documents, of which only 76 met the established criteria and were analyzed in full length.

*Eryngium foetidum* has a high potential for use in different areas of application. It is mainly reported to treat diseases related to the intestinal and gynecological tract, as well as viral diseases and infections. The appreciation of traditional knowledge and eating habits are of inestimable importance to preserve its forms of use and edible biodiversity. Chicory leaves are widely used in food preparation, but few research papers were found on the subject. Thus, there is field for research to be carried out to provide a better understanding of the gastronomic cultural identity involving this species.

The volatile constituents and minerals present in the species have high pharmacological value, with strong antioxidant activity. The essential oils of *E*. *foetidum* are marked by aromatic and aliphatic aldehydes; (2E)-2-dodecenal is the major chemical constituent in the leaves, and 2,3,4-trimethylbenzaldehyde is the major chemical constituent in the roots. These substances are effective to treat diseases and can prevent oxidative deterioration in food. Other constituents such as alkaloids and anthraquinones were found, and these compounds have rarely been found in specimens of *E*. *foetidum* in the last ten years, suggesting that further research is needed on this group and its applications. The pharmacological tests reported in the analyzed works mentioned effective antibacterial, antioxidant, antifungal, and anti-inflammatory actions of *E*. *foetidum* extracts. In this sense, the effectiveness of the use of *E*. *foetidum* in ethnomedicine can be associated with these actions.

Few studies have assessed the toxic, antidiabetic, anticancer, and antilarval potential of *E*. *foetidum*, and thus, further research is needed to support the research already carried out and confirm the effectiveness of the use of this plant to treat other diseases.

## Figures and Tables

**Table 1:** Traditional uses of *Eryngium foetidum* L.

Medicinal	Locality	Popular name	Illness	Plant part used	Form of use	Ref.
	Brazil	Chicória, coentrão	Infection	Leaves	Tea	[39]
			Flu, diarrhea, and stomach pain	Leaves and roots	Tea/syrup	[35]
			Teething, flu, diarrhea	Roots	Tea; tea prepared with chicory root and “sacaca” leaves; tea prepared with chicory root and mint roots	[36]
			Headache		Maceration	[38]
			Cough and urinary infection		—	[55]
			Used to accelerate labor	Roots	Tea	[33]
			Used in religious/cultural rites for “quebranto”	Leaves	Tea/bath	[43]
	Colombia	Cilantro sabanero	Gastrointestinal problems such as flatulence, indigestion, and stomach problems; infections and infestations such as smallpox and worm infestation; respiratory system (flu)		—	[30]
			Hepatitis	Roots	Infusion; boiled and taken three times a day	[41, 42]
			Malaria, seizures, spasms, sexual impotence, gastrointestinal problems (antiflatulent, stomach pain, vomiting, diarrhea, nausea), flu-like symptoms (headache, cough, flu), cramps, bleeding; antiscorbutic, antirheumatic, antiseptic, and febrifuge	Whole plant	Baths and cooked in food	[31]
			Purgative/taenifuge or vermifuge and sedative; used in witchcraft	Leaves	Oral intake at cold temperature; raw	[34]
	Panama	Culantro	Cramps, anxiety, sore throat, and weight loss		—	[37]
	Peru	Siuca culantro	Labor stimulant, cramps, antidiarrheal, menstrual pain, aphrodisiac, abortifacient, diuretic, and antiemetic		—	[28, 32]
	Ecuador	Culantro de monte	Stomach problems such as dysentery, joint pain, especially in the knee	Leaves and roots	Infusion and plaster	[2]
			Skin changes, gastrointestinal and respiratory diseases, pathologies, and disorders of the nervous system	Leaves	—	[40]
	Cameroon	—	Abscesses and boils	Leaves and stems	The leaves are squeezed or ground with a little water resulting to prepare a solution. External use	[17]
	China	—	Cough, dyspepsia (poor digestion), snake bite	—	Decoction, poultice (plaster)	[56]

Gastronomic	Locality		Recipes	Ingredients		Ref.
	Brazil (Pará)		“Caldeirada paraense no tucupi” (Pará stew with tucupi)	Fish, tucupi, green condiments (chicory, scallion, coriander), jambu, garlic, onion, green pepper, bay leaf, basil, tomato, potato, eggs		[9]
			“Pato no tucupi” (duck stew made with tucupi)	Dry salted shrimp, green condiments (chicory, scallion, coriander), okra, jambu, garlic, onion, palm oil, tomato		[9, 52, 54]
			“Caruru”	Dry salted shrimp, green condiments (chicory, scallion, coriander), okra, jambu, garlic, onion, palm oil, tomato		[9]
			“Tacacá”	Chicory, jambu, tucupi, cassava starch, dried shrimp, garlic, and habanero pepper		[9, 51]
			“Mujica”	Cassava flour porridge mixed with crab or fish meat, cooked with lemon in water, garlic, salt, and margarine, seasoned with chicory, basil, and coriander		[7]

**Table 2:** Volatile compounds present in *Eryngium foetidum* L.

Chemical constituents	Leaf (%)	Root (%)	Ref.
(2E)-2-Dodecenal	21.76 ± 30.4	3.75 ± 6.24	[5]
	28.43	7.65	[1]
	43.96	—	[60]
	50.62	—	[61]
	14.3	2.8	[58]
	—	—	[13]
	46.68	—	[14]
13-Tetradecenal	27.45	9.26	[1]
	5.41	—	[60]
*Trans*-2-tetradecenal	8.61 to 13.33	2.46 to 3.75	[5]
Dodecanal	14.59	1.0	[1]
	10.29	—	[61]
	4.7	—	[58]
2,4,6-Trimethylbenzaldehyde	16.5	4.00 and 57.0	
	1.5 and 14.3	2.2 and 24.1	
2,4,5-Trimethylbenzaldehyde	10.77	56.08	[1]
	11.00		[61]
2,3,4-Trimethylbenzaldehyde	19.5 to 24.6	56.81 to 63.49	[5]
5-Dodecene	30.15	—	[60]
Tetradecanal	5.28	—	
3,4,5-Trimethylphenol	3.08	—	
2,4,6-Trimethylbenzaldehyde	2.24	—	
1-(2-Methylbutyl)-1-(1-methylpropyl)cyclopropane	5.94	—	[61]
*α*-Pineno	3.49	—	
(*Z*)-13-octadecenal		—	[62]
Muurola 4,10(14)-dien-1*α*-ol	10.2	—	[58]
Neophytadiene isomer	4.5	—	
Hexahydrofarnesyl acetone	5.5	—	
Neophytadiene isomer	4.5	—	
Hexahydrofarnesyl acetone	5.5	—	
Phytol	4.9	—	
2-Formyl 1,1,5-trimethyl 2,5-cyclohexadien-4-yl-2-methylbutenoate	—	4.9	
*𝜏*-Cadinol	5.1	7.3	
*α*-Cadinol	6.9	10.2	
Caprylic alcohol	14.80	—	[14]
1,4-Dihydrocarbazole-1,4-dione	11.29	—	
Lauraldehyde	10.22	—	
*α*-Pinene	—	—	[62]
M-Cymene	—	—	
O-Cymene	—	—	
Lasidiol *p-*methoxybenzoate	—	—	[63]

**Table 3:** Fixed organic compounds present in *Eryngium foetidum* L.

Chemical constituents	Leaves	Ref.
Chlorogenic acid	4.327 *μ*g/g	[15]
	338 *μ*g/g	[76]
Carbohydrates	174.72 ± 1.72 *μ*g/mg	[75]
	—	[68]
	128.0 ± 5.6 mg/g	[69]
	134.0 ± 5.9 mg/g	
Carotenoids	*μ*g/100 g	[59]
	84.3 ± 3.1 *μ*g/g	[69]
	78.8 ± 5.4 *μ*g/g	
Chlorophyll a	1.32 ± 0.09 mg/g	[77]
	208.9 ± 12.3 *μ*g/g	[69]
	199.5 ± 19.5 *μ*g/g	
Chlorophyll b	0.42 ± 0.03 mg/g	[77]
	101.5 ± 5.3 *μ*g/g	[69]
	93.4 ± 8.3 *μ*g/mg	
Crude fiber	6.32 ± 0.5 *μ*g/mg	[59]
Alkaloids	—	[71]
	365.5 *μ*g/ml	[72]
Phenols	—	[71]
	7.8 ± 0.00 mg/g	[78]
	0.07 ± 0.00 mg/g	
	3.72 ±0.02 mg/g	
	—	[68]
	164600 *μ*g/ml	[72]
	40.4 ± 0.8 mg/g	[69]
	40.4 ± 1.0 mg/g	
	—	[70]
Flavonoids	—	[71]
	—	[12]
	1547.9 *μ*g/ml	[72]
	1.81 ± 0.1 mg/g	[69]
	1.88 ± 0.1 mg/g	
	113.5 ± 180.3 mg/100 g	[16]
	—	[68]
	—	[70]
Anthraquinones	—	[71]
Anthocyanins	19.4 ± 78.9 mg/100 g	[16]
Steroid	—	[71]
		[12]
Glycosides	—	
	—	[68]
Terpenoids	—	[12]
Tannins	—	
	0.04776 *μ*g/ml	[72]
	—	[68]
	76.90°C 88 mg/100 g	[16]
Ascorbic acid	17.1 ± 34.56 mg/g	[78]
	135.2 mg/100 g	[16]
Saponin	255,000 *μ*g/ml	[72]
		[68]
Lutein	692 *μ*g/g	[76]
*𝛽*-carotene	326 *μ*g/g	
Caffeic acid	209 *μ*g/g	
Kaempferol	136 *μ*g/g	
Phytosterol	—	[68]
Gum and mucilages	—	
Reducing sugars	7.67 ± 0.4 mg/g	[69]
	9.53 ± 0.4 mg/g	
Sucrose	54.8 ± 4.1 mg/g	
	71.1 ± 4.7 mg/g	
Polyphenols	217.6 ± 256.7 mg/100 g	[16]

**Table 4:** Pharmacological activities of bioactive compounds from *Eryngium foetidum* L.

Pharmacological activity	Organism	Bioactive compounds	Ref.
Antibacterial and antifungal	*Helicobacter pylori*	Alkaloids, phenols, flavonoids, anthraquinone, sterols	[71]
	*Streptococcus pneumoniae*, *Listeria monocyatogenes*, *Staphylococcus aureus,* and *Salmonella*	Glycosides, flavonoids, terpenoids, steroids, and tannins	[12]
		Phenolic compounds, ascorbic acid	[78]
	*Salmonella typhimurium* and *Candida albicans*	Flavonoids, tannins, alkaloids, phenolic compounds, steroids, and terpenoids	[72]
	*Escherichia coli*, *Pseudomonas aeruginosa*, *Staphylococcus aureus subsp*. *aureus* e *Streptococcus pneumoniae*	—	[80]
	*Bacillus cereus*, *Staphylococcus aureus*, *Escherichia coli*, *Salmonella*, *Shigella sonnei*, *Shigella dysenteriae*, *Shigella flexneri,* and *Vibrio cholerae MTCC*	—	[79]

Anthelmintic	*Strongyloides stercoralis*	Trans-2-dodecenal	[13]
Anti-leishmaniasis	*Leishmania tarentolae* promastigotes and *Leishmania donovani* amastigotes	Lasidiol *p-*methoxybenzoate	[63]
Antilarval	*Aedes albopictus skuse*	2-Dodecen-1-al, capryl alcohol	[14]
Anti-inflammatory	—	Lutein, *β*-carotenes, chlorogenic acid, kaempferol, and caffeic acid	[81]
		*β*-Carotene, lutein, caffeic acid, and kaempferol	[76]
Anticlastogenic	—	—	[82]
Anticancer	—	—	[83]
Antidiabetic	—	Carbohydrates, starch, mucilage, proteins and amino acids, saponins, phytosterols, flavonoids, phenolic compounds, and tannins	[68]

Antioxidant	—	Polyphenols, flavonoids, chlorophylls, and carotenoids	[69]
		carotenoids (*β*-carotene, *β*-cryptoxanthin, lutein, zeaxanthin, pheophytin-b, chlorophyll-a, and chlorophyll-b), phenolic compounds ( protocatechuic acid, p-coumaric acid, syringic acid ferulic acid, and sinapic acid, gallic acid) and anthroquinones (citreorosein, telochistin, secalonic acid D, emodin, parietin and norlichexanthone).	[16]
		*α*-Pinene, M-cymene, O-cymene, and (Z)-13-octadecenal	[62]
		Phenols, flavonoids, and antioxidants	[70]

Toxicological	Causes renal dysfunction in mice, with a diet of 0.8% of consumption, equivalent to 35% of human consumption in 24 weeks	—	[84]

## Data Availability

All supporting data are included within the article.
